# Distribution of Spoligotyping Defined Genotypic Lineages among Drug-Resistant *Mycobacterium tuberculosis* Complex Clinical Isolates in Ankara, Turkey

**DOI:** 10.1371/journal.pone.0030331

**Published:** 2012-01-18

**Authors:** Ozgul Kisa, Gulnur Tarhan, Selami Gunal, Ali Albay, Riza Durmaz, Zeynep Saribas, Thierry Zozio, Alpaslan Alp, Ismail Ceyhan, Ahmet Tombak, Nalin Rastogi

**Affiliations:** 1 Department of Medical Microbiology, Gulhane Military Medical Academy and School of Medicine, Ankara, Turkey; 2 Tuberculosis Reference and Research Laboratory, Refik Saydam Hygiene Center, Ankara, Turkey; 3 Inonu University Faculty of Medicine, Department of Medical Microbiology, Inonu University, Malatya, Turkey; 4 Hacettepe University Faculty of Medicine, Department of Medical Microbiology, Hacettepe University, Ankara, Turkey; 5 WHO Supranational TB Reference Laboratory, Unité de la Tuberculose et des Mycobactéries, Institut Pasteur de Guadeloupe, Abymes, Guadeloupe, France; University of Cape Town, South Africa

## Abstract

**Background:**

Investigation of genetic heterogeneity and spoligotype-defined lineages of drug-resistant *Mycobacterium tuberculosis* clinical isolates collected during a three-year period in two university hospitals and National Tuberculosis Reference and Research Laboratory in Ankara, Turkey.

**Methods and Findings:**

A total of 95 drug-resistant *M. tuberculosis* isolates collected from three different centers were included in this study. Susceptibility testing of the isolates to four major antituberculous drugs was performed using proportion method on Löwenstein–Jensen medium and BACTEC 460-TB system. All clinical isolates were typed by using spoligotyping and IS*6110*-restriction fragment length polymorphism (RFLP) methods. Seventy-three of the 95 (76.8%) drug resistant *M. tuberculosis* isolates were isoniazid-resistant, 45 (47.4%) were rifampicin-resistant, 32 (33.7%) were streptomycin-resistant and 31 (32.6%) were ethambutol-resistant. The proportion of multidrug-resistant isolates (MDR) was 42.1%. By using spoligotyping, 35 distinct patterns were observed; 75 clinical isolates were grouped in 15 clusters (clustering rate of 79%) and 20 isolates displayed unique patterns. Five of these 20 unique patterns corresponded to orphan patterns in the SITVIT2 database, while 4 shared types containing 8 isolates were newly created. The most prevalent *M. tuberculosis* lineages were: Haarlem (23/95, 24.2%), ill-defined T superfamily (22/95, 23.2%), the Turkey family (19/95, 20%; previously designated as LAM7-TUR), Beijing (6/95, 6.3%), and Latin-America & Mediterranean (LAM, 5/95 or 5.3%), followed by Manu (3/95, 3.2%) and S (1/95, 1%) lineages. Four of the six Beijing family isolates (66.7%) were MDR. A combination of IS*6110*-RFLP and spoligotyping reduced the clustering rate from 79% to 11.5% among the drug resistant isolates.

**Conclusions:**

The results obtained showed that ill-defined T, Haarlem, the Turkey family (previously designated as LAM7-TUR family with high phylogeographical specifity for Turkey), Beijing and LAM were predominant lineages observed in almost 80% of the drug-Resistant *M. tuberculosis* complex clinical isolates in Ankara, Turkey.

## Introduction

Tuberculosis (TB) continues to be one of the prevalent infectious diseases. The global spread of the disease is further complicated by the ubiquitous appearance of drug resistant and especially MDR isolates (the latter being defined by combined resistance to isoniazid [INH] and rifampicin [RIF]) [1], [2]. One of the greatest concerns of TB control programs is the emergence and spread of drug resistant and MDR-TB. According to a recent report of the Ministry of Health [3], with an incidence rate of 30 per 100,000 inhabitants, there were 16.760 new TB cases in Turkey in 2008 while the total number of TB cases was 18.452. The available figures for Ankara for the same year reported a total of 665 TB cases (55.8% male, 44.2% female, sex-ratio 1.26), with smear and culture positivity rates of 56.2% and 71.4%, respectively. The resistance proportion to isoniazid, rifampicin, ethambutol, streptomycin, and MDR were 13.8%, 6.6%, 4.3%, 7.5%, and 5.3% respectively [3]. According to the results of the previous studies from different parts of Turkey, the resistance rates to at least one drug ranged from 14.9% to 40.2% [4]–[7]. Also, MDR rates were between 1.7% and 19.7% [4]–[6].

Study of genotyping diversity of drug resistant *M. tuberculosis* isolates from infected individuals can provide useful information about the origin and transmission of the drug resistant isolates [8]–[11]. Although the rates of drug resistant *M. tuberculosis* isolates are high in Turkey, the information about resistant *M. tuberculosis* genotypes circulating in the country is very limited. Despite several publications dealing with *M. tuberculosis* genotyping in Turkey [1], [8], [12]–[16], only two investigations reported spoligotyping-based characterization of drug resistant *M. tuberculosis* isolates [1], [8]. This study was therefore planned to estimate the clustering rate and pinpoint precisely the prevalent lineages among the drug resistant *M. tuberculosis* isolates in Ankara, the capital city of Turkey. For this purpose we used spoligotyping and IS*6110*-RFLP that are two well-established methods used frequently to highlight transmission dynamics and phylogenetic distribution of *M. tuberculosis* isolates all over the world [10], [17]–[19]. PCR-based spoligotyping is rapid, cost-effective, and allows concomitantly to differentiate mycobacterial species within the *M. tuberculosis* complex as well as to easily identify specific genotypes such as those belonging to the Beijing lineage [20], while IS*6110*-RFLP allows to substantially reduce the overestimation of clustered *M. tuberculosis* isolates by spoligotyping used alone as a first-line typing method [12].

## Materials and Methods

### Patient population and bacterial isolates

Our analysis was based on 95 *M. tuberculosis* isolates from various clinical samples from 2004 to the end of 2006 (1 isolate per patient; n = 35 from Mycobacteriology laboratory of Gulhane Military Medical Academy, n = 33 from Hacettepe University, and n = 27 from National TB Reference and Research Laboratory in the Refik Saydam Hygiene Center, Ankara). The ages of the 95 patients ranged from 19 to 70 years, with a mean of 38.12±19.18. It should be noted that all *M. tuberculosis* isolate found to be drug-resistant from the study laboratories over the study period were included in this study and no other selection criteria than drug-resistance were retained. More specifically, due to a lack of systematic sampling in our region, it is difficult to interpret our study sample in terms of representativity in a national context; hence we have arbitrarily decided to designate it as a “convenience sample”.

### Identification and drug susceptibility testing

Differentiation of the *M. tuberculosis* complex and non-tuberculous mycobacteria was achieved by selective inhibition of the *M. tuberculosis* isolates in the presence of 5 µl/ml of p-nitro-α-acetyl-amino-β-hydroxypropiophenone (NAP) according to the BACTEC manual [21]. All isolates grown on LJ media were identified as *M. tuberculosis* by using biochemical tests, including production of niacin, catalase activity, nitrate reduction, pigment production and growth rate [22]. Drug susceptibility testing against rifampicin (R), isoniazid (I), streptomycin (S), and ethambutol (E) was performed by using the BACTEC 460-TB according to the manufacturer's instruction or standard proportion method on LJ medium [21], [23]. Multidrug-resistant (MDR) clinical isolates were defined as simultaneous resistance to I+R, with or without additional drug resistance.

Verbal consent for diagnosis of TB and detection of drug resistance in *M. tuberculosis* isolates were obtained from all patients. Nonetheless, since the resistant isolates were collected from patients' routine samples, this study was considered as a laboratory study and ethics approval from institutional ethics committee was not required.

### DNA extraction

A loopful of bacteria colonies was suspended in 400 µl 1× TE buffer (10 mM Tris, 1 mM EDTA, pH 8.0), and inactivated at 80°C for 20 minutes. Bacterial DNA was extracted by the standard cetyl-trimethyl ammonium bromide (CTAB) (Merck, Darmstadt, Germany) method, as described previously [24]. The pellet of DNA was dried at room temperature, resuspended in 1× TE buffer and stored at 4°C until use.

### Spoligotyping

Spoligotyping was carried out with a commercial kit (Isogen Bioscience BV, Maarssen, The Netherlands) according to the manufacturer's instructions [11]. The 43 spacers between the direct repeats in the target region were amplified by using DRa biotinylated at the 5′ end and DRb primers. The PCR product was hybridized to a membrane containing 43 oligonucleotides derived from the spacer sequences of *M. tuberculosis* H37Rv and *M.bovis* BCG P3 by reverse line blotting. *M. tuberculosis* H37Rv and *M.bovis* BCG P3 were used as control for spoligotyping. Spoligotypes results were converted into octal code and in the SITVIT2 proprietary database of the Pasteur Institute of Guadeloupe, which is an updated version of the previously released SpolDB4 database (available at http://www.pasteur-guadeloupe.fr:8081/SITVITDemo). At the time of the present study, the database contained genotyping information on about 67,000 *M. tuberculosis* clinical isolates from 160 countries of origin. In this database, spoligotype international type (SIT) designates a spoligotyping pattern shared by two or more patient isolates. Major spoligotyping-based phylogenetic clades were assigned according to signatures provided in SpolDB4.

### IS*6110*-RFLP typing

*IS6110*-RFLP genotyping was performed by the standardized methods described previously [25]. Briefly, the extracted DNA of clinical isolates was digested with PvuII enzyme and DNA fragments were subjected to electrophoresis. The restriction fragments on the gel were denatured, blotted onto nylon membrane by the alkaline transfer procedure and hybridized by IS*6110* probe. After hybridization, the results were analyzed by using H37Rv as an internal marker, and comparison was done using Bionumerics (Applied Maths, Sint-Martens-Latem, Belgium).

## Results

### Drug susceptibility

All of the *M. tuberculosis* isolates were resistant to at least one of the major antituberculous drugs. Seventy-three of the 95 *M. tuberculosis* isolates (76.8%) were resistant to isoniazid, 45 (47.4%) were resistant to rifampicin, 32 (33.7%) were resistant to streptomycin and 31 (32.6%) were resistant to éthambutol, and the proportion of MDR isolates was 42.1% (Table 1).

**Table 1:** Drug resistance patterns of the 95 drug-resistant *M. tuberculosis* isolates from Ankara, Turkey.

Drugs	Number of the resistant isolatesn (%)
Streptomycin (S)	32(33.6)
Isoniazid (I)	73 (76.8)
Rifampicin (R)	45 (47.4)
Ethambutol (E)	31 (32.6)
S+I	12 (12.6)
I+E	2 (2.1)
R+E	2 (2.1)
S+I+R	5 (5.3)
I+R+E	14 (14.7)
S+I+R+E	10 (10.5)
Total MDR (I+R)	40 (42.1)
Total	95

### Spoligotyping

**Figure 1:**
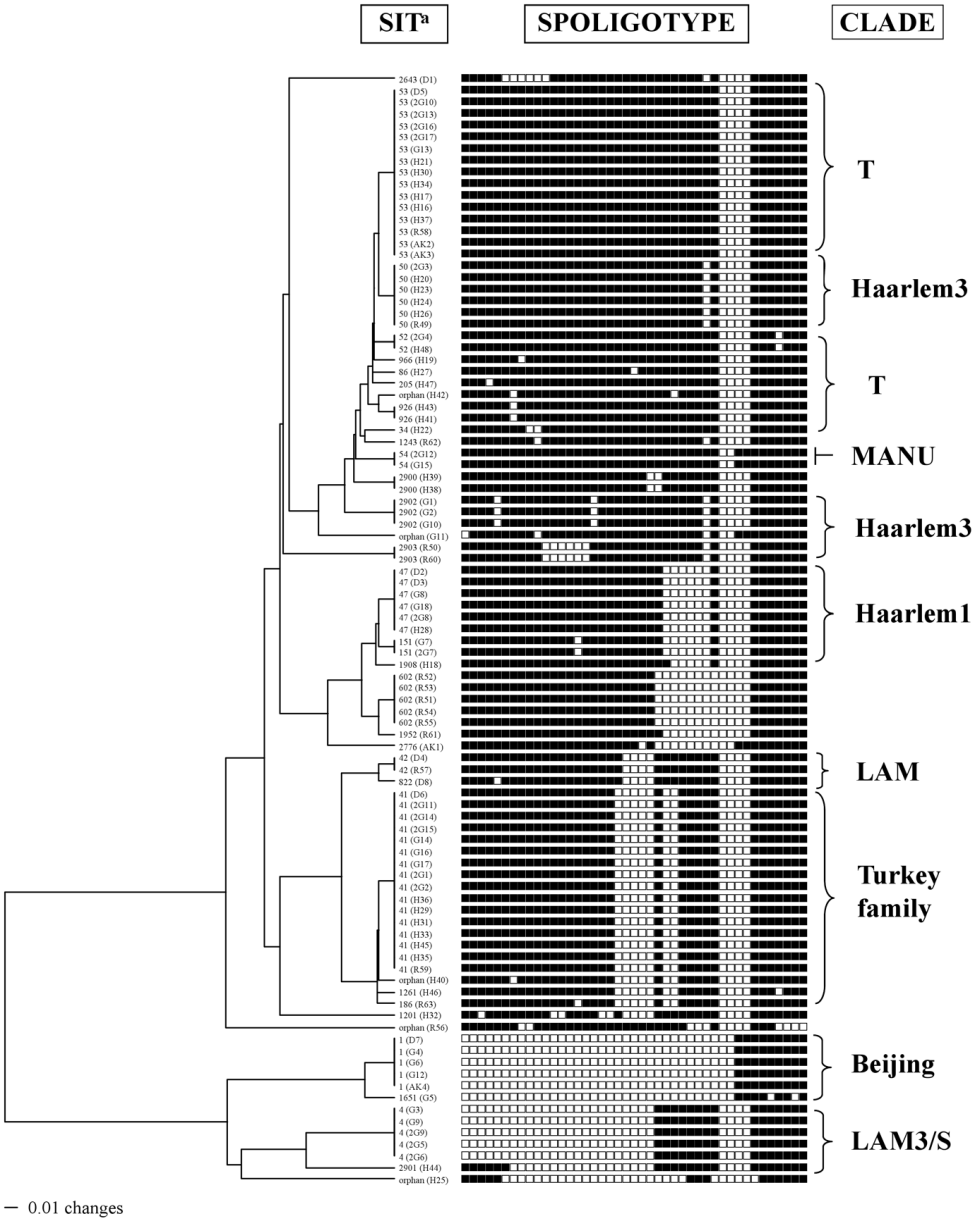
Tree of spoligotype *M. tuberculosis* isolates (n = 95 strains), by using the PAUP 4.0b software and the UPGMA method. *^a^*SIT, Spoligotype International Type as defined in the SITVIT2 proprietary database of the Pasteur Institute of Guadeloupe. In the figure, the column below the subtitle SIT shows the individual SIT number followed by strain number.

Spoligotyping of the 95 *M. tuberculosis* isolates yielded 35 different patterns, 20 of these patterns were unique (1 isolate only), whereas 15 patterns containing 75 isolates were clustered (2 to 16 isolates per cluster, clustering rate of 79%, note that the detailed spoligotyping results are summarized in Table 2, and the UPGMA tree illustrated in Figure 1). Ninety of the 95 *M. tuberculosis* isolates belonged to 30 shared types (SITs), 4 of these SITs containing 8 isolates were newly created in the database, whereas 26 SITs containing 82 isolates matched preexisting patterns. Regarding 4 newly-created SITs, 2 included isolates unique to this study, 1 matched with an orphan from Italy, and another an orphan from the United States. Regarding the distribution of shared types, SIT41 (Turkey family) predominated in our setting with 19/95 (20%) of the isolates, and was followed by SIT53 (ill-defined T) with 15/95 or 15.8% of isolates, SIT47 and SIT50 with 6/95 (6.3%) of the isolates, SIT1 and SIT4 with 5/95 (5.3%) of the isolates, and other less frequent SITs representing 1–3 isolates (Table 2).

**Table 2:** Description of 30 shared types containing 90 isolates that matched a preexisting shared type in the SITVIT2 proprietary database of the Pasteur Institute of Guadeloupe.

SIT	Spoligotype Description	Number in study	% in study	% in Database	% in study vs. Database^a^	Lineage^b^	Match with an orphan from^c^
1	□□□□□□□□□□□□□□□□□□□□□□□□□□□□□□□□□□▪▪▪▪▪▪▪▪▪	5	5.26	10.62	0.08	Beijing	
4	□□□□□□□□□□□□□□□□□□□□□□□□▪▪▪▪▪▪▪▪□□□□▪▪▪▪▪▪▪	5	5.26	0.42	2.07	LAM3/S	
34	▪▪▪▪▪▪▪▪□□▪▪▪▪▪▪▪▪▪▪▪▪▪▪▪▪▪▪▪▪▪▪□□□□▪▪▪▪▪▪▪	1	1.05	1.13	0.16	S	
41	▪▪▪▪▪▪▪▪▪▪▪▪▪▪▪▪▪▪▪□□□□□▪□□▪▪▪▪▪□□□□▪▪▪▪▪▪▪	16	16.84	0.51	5.48	Turkey	
42	▪▪▪▪▪▪▪▪▪▪▪▪▪▪▪▪▪▪▪▪□□□□▪▪▪▪▪▪▪▪□□□□▪▪▪▪▪▪▪	2	2.11	3.76	0.09	LAM	
47	▪▪▪▪▪▪▪▪▪▪▪▪▪▪▪▪▪▪▪▪▪▪▪▪▪□□□□□□▪□□□□▪▪▪▪▪▪▪	6	6.32	1.91	0.55	Haarlem1	
50	▪▪▪▪▪▪▪▪▪▪▪▪▪▪▪▪▪▪▪▪▪▪▪▪▪▪▪▪▪▪□▪□□□□▪▪▪▪▪▪▪	6	6.32	4.13	0.26	Haarlem3	
52	▪▪▪▪▪▪▪▪▪▪▪▪▪▪▪▪▪▪▪▪▪▪▪▪▪▪▪▪▪▪▪▪□□□□▪▪▪□▪▪▪	2	2.11	0.99	0.36	T	
53	▪▪▪▪▪▪▪▪▪▪▪▪▪▪▪▪▪▪▪▪▪▪▪▪▪▪▪▪▪▪▪▪□□□□▪▪▪▪▪▪▪	15	15.79	7.12	0.37	T	
54	▪▪▪▪▪▪▪▪▪▪▪▪▪▪▪▪▪▪▪▪▪▪▪▪▪▪▪▪▪▪▪▪□□▪▪▪▪▪▪▪▪▪	2	2.11	0.22	1.59	Manu	
86	▪▪▪▪▪▪▪▪▪▪▪▪▪▪▪▪▪▪▪▪▪□▪▪▪▪▪▪▪▪▪▪□□□□▪▪▪▪▪▪▪	1	1.05	0.12	1.45	T	
151	▪▪▪▪▪▪▪▪▪▪▪▪▪▪□▪▪▪▪▪▪▪▪▪▪□□□□□□▪□□□□▪▪▪▪▪▪▪	2	2.11	0.04	9.09	Haarlem1	
186	▪▪▪▪▪▪▪▪▪▪▪▪▪▪□▪▪▪▪□□□□□▪□□▪▪▪▪▪□□□□▪▪▪▪▪▪▪	1	1.05	0.02	9.09	Turkey	
205	▪▪▪□▪▪▪▪▪▪▪▪▪▪▪▪▪▪▪▪▪▪▪▪▪▪▪▪▪▪▪▪□□□□▪▪▪▪▪▪▪	1	1.05	0.06	3.13	T	
602	▪▪▪▪▪▪▪▪▪▪▪▪▪▪▪▪▪▪▪▪▪▪▪▪□□□□□□□□□□□□▪▪▪▪▪▪▪	5	5.26	0.14	6.10	Unk	
822	▪▪▪▪□▪▪▪▪▪▪▪▪▪▪▪▪▪▪▪□□□□▪▪▪▪▪▪▪▪□□□□▪▪▪▪▪▪▪	1	1.05	0.03	5.88	LAM	
926	▪▪▪▪▪▪□▪▪▪▪▪▪▪▪▪▪▪▪▪▪▪▪▪▪▪▪▪▪▪▪▪□□□□▪▪▪▪▪▪▪	2	2.11	0.03	11.76	T	
966	▪▪▪▪▪▪▪□▪▪▪▪▪▪▪▪▪▪▪▪▪▪▪▪▪▪▪▪▪▪▪▪□□□□▪▪▪▪▪▪▪	1	1.05	0.02	8.33	T	
1201	▪▪□▪▪▪▪▪▪▪▪□□▪▪▪▪□□▪□□□□▪▪▪▪▪▪▪▪□□□□▪▪▪▪▪▪▪	1	1.05	0.01	25.00	LAM	
1243	▪▪▪▪▪▪▪▪▪□▪▪▪▪▪▪▪▪▪▪▪▪▪▪▪▪▪▪▪▪□▪□□□□▪▪▪▪▪▪▪	1	1.05	0.02	9.09	Haarlem3	
1261	▪▪▪▪▪▪▪▪▪▪▪▪▪▪▪▪▪▪▪□□□□□▪□□▪▪▪▪▪□□□□▪▪▪□▪▪▪	1	1.05	0.04	4.17	Turkey	
1651	□□□□□□□□□□□□□□□□□□□□□□□□□□□□□□□□□□▪▪▪▪□▪▪□▪	1	1.05	0.01	25.00	Beijing	
1908	▪▪▪▪▪▪▪▪▪▪▪▪▪▪▪▪▪▪▪▪▪▪▪▪▪▪□□□□□▪□□□□▪▪▪▪▪▪▪	1	1.05	0.01	20.00	Haarlem1	
1952	▪▪▪▪▪▪▪▪▪▪▪▪▪▪▪▪▪▪▪▪▪▪▪▪▪□□□□□□□□□□□▪▪▪▪▪▪▪	1	1.05	0.02	8.33	Unk	
2643	▪▪▪▪▪□□□□□□▪▪▪▪▪▪▪▪▪▪▪▪▪▪▪▪▪▪▪□▪□□□□▪▪▪▪▪▪▪	1	1.05	0.02	7.14	Haarlem3	
2776	▪▪▪▪▪▪▪▪▪▪▪▪▪▪▪▪▪▪▪▪▪▪□▪□□□□□□□□□□▪▪▪▪▪▪▪▪▪	1	1.05	0.01	33.33	Unk	
2900	▪▪▪▪▪▪▪▪▪▪▪▪▪▪▪▪▪▪▪▪▪▪▪□□▪▪▪▪▪▪▪□□□□▪▪▪▪▪▪▪	2	2.11	New SIT	New SIT	Unk	Italy
2901	▪▪▪▪▪▪□□□□□□□□□□□□□□□□□□▪▪▪▪▪▪▪▪□□□□▪▪▪▪▪▪▪	1	1.05	New SIT	New SIT	LAM	USA
2902	▪▪▪▪□▪▪▪▪▪▪▪▪▪▪▪□▪▪▪▪▪▪▪▪▪▪▪▪▪□▪□□□□▪▪▪▪▪▪▪	3	3.16	New SIT	New SIT	Haarlem3	This study
2903	▪▪▪▪▪▪▪▪▪▪□□□□□□▪▪▪▪▪▪▪▪▪▪▪▪▪▪□▪□□□□▪▪▪▪▪▪▪	2	2.11	New SIT	New SIT	Haarlem3	This study

a The percentage in this study as compared to the SITVIT2 database was calculated by dividing the number of strains with a given pattern in the present study by the total number of strains with the same pattern in the database, and then multiplying the figure by 100 to get the percentage with respect to the total amount. Note that SITVIT2 is an updated version of the previously released SpolDB4 database [27]. At the time of the present analysis, it contained genotyping information on 67,000 *M. tuberculosis* clinical isolates from 160 countries of patient origin.

b Lineage designation, LAM: Latin-American and Mediterranean; Unk: Unknown patterns within any of the major lineage described in database.

c For newly created SITs, match with an orphan isolate is shown in the database.

Thus the major lineages observed ranked in the following order: Haarlem, 23/95 or 24.2%; ill-defined T superfamily (not a lineage *sensu stricto* since it is defined by default), 22/95 or 23.2%; the Turkey family (19/95, 20%; previously designated as LAM7-TUR), Beijing, 6/95 or 6.3%; LAM (Latin-America & Mediterranean), 5/95 or 5.3%; Manu, 3/95 or 3.2%; S, 1/95 or 1%. Comparison of the spoligotyping results with the updated SITVIT2 database revealed that 5 patterns did not match any existing isolate, and these were designated as orphans (Table 3). Of the 40 MDR isolates, 18 (45%) corresponded to unique patterns within this study as opposed to 22 (55%) clustered isolates (6 clusters containing 2 to 8 isolates per cluster). The distribution of lineages among MDR isolates was as follows: ill-defined T superfamily (12/40, 30%), Haarlem (8/40, 20%), the Turkey family (which was previously labeled as LAM7-TUR; 5/40, 12.5%), Beijing (4/40, 10%), LAM (3/40, 7.5%), and Manu (1/40, 2.5%), lineages.

**Table 3:** Description of the orphan isolates (n = 5) after comparison of the spoligotype patterns of the drug-resistant *M. tuberculosis* isolates from Ankara with the SITVIT2 database.

Iso Number	Spoligotype Description	Octal Number	Lineage
TUR062005300G11	□▪▪▪▪▪▪▪▪□▪▪▪▪▪▪▪▪▪▪▪▪▪▪▪▪▪▪▪▪□▪□□▪▪▪▪▪▪▪▪▪	377377777723771	Manu
TUR082005200H25	▪▪▪▪▪□□□□□□□□□□□□□□□□□□□□□□□▪▪▪□□□□□□▪▪▪▪▪▪	760000000340371	Unknown
TUR082004200H40	▪▪▪▪▪▪□▪▪▪▪▪▪▪▪▪▪▪▪□□□□□▪□□▪▪▪▪▪□□□□▪▪▪▪▪▪▪	773777404760771	Turkey
TUR082004200H42	▪▪▪▪▪▪□▪▪▪▪▪▪▪▪▪▪▪▪▪▪▪▪▪▪▪□▪▪▪▪▪□□□□▪▪▪▪▪▪▪	773777776760771	Unknown
TUR092005300R56	▪▪▪▪▪▪▪□□▪▪▪▪▪▪▪▪▪▪▪▪▪▪▪▪▪▪▪□□□▪□□□□▪▪▪□□□□	774777777420700	Haarlem

### IS*6110*-RFLP typing

IS*6110*-RFLP typing of 87 isolates (because of culture contamination, 8 isolates were not typed by IS*6110*-RFLP) identified 76 fingerprint patterns, 7 were clustered including 18 isolates with a clustering rate of 20.7%, the remaining 69 were unique. The number of IS*6110* copies varied from 2 to 14; 29.9% of the isolates were low-copy-number isolates having less than six copies of IS*6110*. A combination of IS*6110*-RFLP and spoligotyping reduced the number of clustered isolates to 10 and the clustering rate to 11.5%.

## Discussion

Antituberculous drug resistance, especially MDR is a major factor threatening the success of TB control programs [26]. In this context, the molecular typing of *M. tuberculosis* clinical isolates provides with useful data about success of TB control and treatment protocols in a given population [16], [27]. By calculating the rate of clustering, efficacy of TB control program in a study population can be estimated [16]. Indeed, it is generally accepted that clustering indicates ongoing or recent transmission, while unique patterns indicate reactivation events [16], [28]. In this study, spoligotyping of the 95 drug-resistant *M. tuberculosis* clinical isolates resulted in a very high clustering rate (79%). This clustering rate was similar to the results of the previous studies used spoligotyping (77%, 76%, 85%) in Turkey [8], [13], [14]. These rates are closer to the rates reported in Harare, Zimbabwe (84.1%) and in Finland (90%), but much higher than the rates of clustered drug-resistance cases observed in studies performed in Sweden (23.6%) and India (43.5%) [9], [17], [18], [29]. Analysis of the resistance patterns revealed that 42% of patients in our setting were infected with MDR isolates, divided in 6 different clusters belonging to LAM, the Turkey family, Beijing, H1, H3 and T (a clustering rate of 55%). These results seem to argue in favor of inadequate monitoring and control of drug resistant TB in Ankara. Nonetheless, since spoligotyping used alone may overestimate the proportion of clustered *M. tuberculosis* isolates as compared to IS*6110*-RFLP and/or MIRU-VNTR, we further evaluated these typing results by spoligotyping and IS*6110*-RFLP used in combination, which reduced the clustering rate dramatically from 79% to 11.5% in our study population.

Major spoligotyping-based lineages of *M. tuberculosis* clinical isolates from different geographical areas of Turkey were assessed in previous studies [8], [14], [16]. Two of these studies showed that LAM7-TUR lineage corresponded to the most frequent spoligotype pattern SIT41 (21% and 22.5%, respectively) in the respective study populations [8], [16]; which allowed the description of a new phylogeographically-specific clone of *M. tuberculosis*, designated LAM7-TUR [16]. In another study from Ankara performed on 114 isolates, the frequency of this spoligotype was 7.9% and it was the 2^nd^ most prevalent spoligotype pattern [14]. A retrospective analysis of a paper on 374 isolates from Turkey published in 2005, i.e., a year before the designation LAM7-TUR was officially published, showed that as high as 14.4% of *M. tuberculosis* isolates belonged to LAM7-TUR which stood as the 2^nd^ most common spoligotype [13]. However, since the description of this *M. tuberculosis* clone with high phylogenetical specificity for Turkey, a recent paper showed that although LAM7-TUR isolates have a spoligotype pattern very close to LAM lineage, they do not belong to the LAM lineage sensu-stricto [30]; instead these strains share a SNP ligC (809 tgg [trp] to ttg [leu]) with the T3-Osaka lineage [30]. Nonetheless, these new results do not question the phylogeographical specificity of this lineage defined by its prototype SIT41 in the SpolDB4 database [27]. Awaiting a detailed molecular characterization of this unique lineage in future studies, we have hereby renamed the LAM7-TUR lineage as the “Turkey” family.

The current study also showed that 19/95 or 20% of the drug-resistant isolates belonged to the Turkey family, and represented 5/40 or 12.5% of the MDR isolates. The proportion of drug-resistant versus MDR isolates for other major lineages was: Haarlem (24.2% vs. 20%), ill-defined T superfamily (23.2% vs. 30%), Beijing (6.3% vs. 10%), Manu (3.2% vs. 2.5%) and S (1% vs. 0%) lineages. However, if the percentage of MDR isolates was considered within each of the families individually, the order was as follows: Beijing (4/6, 66.7%), LAM (3/5, 60%), T (12/22, 54.5%), Haarlem (8/23, 34.8%), Manu (1/3, 33.3%), the Turkey family (5/19, 26.3%), and S (0/1, 0%).

According to the previous studies performed in Turkey, the four most prevalent spoligotype patterns in Turkey were: ill-defined T/SIT53, Turkey family/SIT41, H1/SIT47, and H3/SIT50 [8], [13], [14], [16]. Although, additional analysis of isolates from various regions of Turkey will be necessary to determine the actual prevalence of these genotypes, our study corroborates these findings and indicates that the Turkey family can be considered as a major type with demonstrated phylogeographical specificity for Turkey as opposed to SIT47, SIT50 and SIT53 that are distributed equally in Turkey and elsewhere [10], [20], [30], [31]. Indeed, a cross-checking of the SITVIT2 database on November 24^th^ 2011 showed that Turkey/SIT41 isolates in the database (n = 357) are almost exclusively isolated in Turkey (n = 212 or 59.38% of the isolates). The results of this study also revealed 4 newly-created shared types (SIT2900, SIT2901, SIT2902 and SIT2903) and 2 of these containing 5 strains were exclusively found in the present study (SIT2902, SIT2903; Table 2). Coupled to the observation of 5 orphan clinical isolates (Table 3), our study indicates that the bulk of TB in Ankara is caused both by strains that are restricted to our local population (as reflected by newly-created shared types or orphans restricted to Ankara as well as endemic strains such as SIT41 belonging to the Turkey family) and those that are present elsewhere (SIT47, SIT50 and SIT53) [8], [14], [16].

Beijing genotype of *M. tuberculosis* has been observed to be highly prevalent throughout Asia and in the countries of the former Soviet Union [32]. Although the Beijing genotype was found only in 6/95 or 6.3% isolates in our study, it corresponded to the lineage with the highest proportion of MDR isolates since 4/6 isolates or 66.7% were MDR. As summarized in Table 4, the relative occurrence of Beijing genotype at 6.3% in the present study was significantly higher than in previous studies from Turkey (0.5%, 1.7%, and 1.8%, respectively) [8], [13], [14]. Seeing the sample size, our attempts to compare lineages in case of MDR versus monoresistant isolates did not result in any statistically significant differences. For the same reason, one may be cautious not to overestimate the prevalence of the Beijing genotype and its probable role in current MDRTB transmission; nonetheless, these observations should not be overlooked while considering the emergence and spread of drug resistant and MDR-TB by the national TB control program.

**Table 4:** Statistical comparison of Beijing isolates in this study and in the previously published studies from Turkey.

	Reference	Beijingn (%)	Other lineagesn (%)	Chi-2	p Value
This study	-	6 (6.3%)	89 (93.7%)	11.06	0.01
Durmaz et al., 2007	[8]	1 (0.5%)	199 (99.5%)		
Gencer et al., 2005	[13]	2 (1.7%)	114 (98.3%)		
Kisa et al., 2007	[14]	7 (1.8%)	360 (98.1%)		

In conclusion, this study showed that the 5 major lineages observed among the drug-resistant *M. tuberculosis* clinical isolates in Ankara were: Haarlem 24.2%; ill-defined T superfamily 23.2%; the Turkey family (previously designated as LAM7-TUR) 20%; Beijing 6.3%; and LAM 5.3. A high proportion of these strains (42.1%) corresponded to MDR isolates with the following main lineages: T 30%; Haarlem 20%; the Turkey family 12.5%; Beijing 10%; and LAM 7.5. However, the order was inversed if the percentage of MDR isolates was calculated within a genotypic lineage individually as: Beijing 66.7%; LAM 60%; T 54.5%; Haarlem 34.8%; Manu 33.3%; and the Turkey family 26.3%. We showed that the bulk of drug-resistant TB in Ankara is caused both by strains that are endemic to the local population (as reflected by newly-created shared types or orphans restricted to Ankara as well as endemic strains such as SIT41 belonging to the Turkey family) and those that are present elsewhere in the world (SIT47, SIT50 and SIT53). Last but not least, the role of Beijing genotype in current MDR-TB transmission in Ankara and elsewhere in Turkey must be reassessed in future studies.
